# Economic Policy Uncertainty, Environmental Regulation, and Green Innovation—An Empirical Study Based on Chinese High-Tech Enterprises

**DOI:** 10.3390/ijerph18189503

**Published:** 2021-09-09

**Authors:** Yue Zhu, Ziyuan Sun, Shiyu Zhang, Xiaolin Wang

**Affiliations:** 1 School of Economics and Management, China University of Mining and Technology, Xuzhou 221116, China; angirl820@126.com (Y.Z.); 13340902339@163.com (S.Z.); ts19070160a31@cumt.edu.cn (X.W.); 2 College of Economics and Management, Huaiyin Normal University, 71 Jiaotong Avenue, Huaian 223300, China

**Keywords:** environmental regulation, green innovation, economic policy uncertainty, moderating effect

## Abstract

As the continuous changes in environmental regulations have a non-negligible impact on the innovation activities of micro subjects, and economic policy uncertainty has become one of the important influencing factors to be considered in the development of enterprises. Therefore, based on the panel data of Chinese high-tech enterprises from 2012–2017, this paper explores the impact of heterogeneous environmental regulations on firms’ green innovation from the perspective of economic policy uncertainty as a moderating variable. The empirical results show that, first, market-incentivized environmental regulation instruments have an inverted U-shaped relationship with innovation output, while voluntary environmental regulation produces a significant positive impact. Second, the U-shaped relationship between market-based environmental regulation and innovation output becomes more pronounced when economic policy uncertainty is high. However, it plays a negative moderating role in regulating the relationship between voluntary-based environmental regulation and innovation output. This paper not only illustrates the process of technological innovation by revealing the intrinsic mechanism of environmental regulation on firm innovation, but also provides insights for government in environmental governance from the perspective of economic policy uncertainty as well.

## 1. Introduction

In recent years, as China’s economic construction has entered a stage of high-quality development and the construction of ecological civilization has continued to advance, people’s green concept has been continuously enhanced. At the same time, Chinese enterprises are also facing the challenge of green transformation and upgrading to contribute the harmonious development. Promoting the upgrading of industrial structure in order to transform the driving force of economic growth has become an important way of economic development in the new era.

In China’s environmental governance, the main role is still played by local governments, and the effectiveness of environmental regulations in each region directly determines the effectiveness of environmental governance at the macro level in China (Wang and Liu 2020) [1]. With the continuous improvement of environmental regulation policy system, the design of environmental regulation tools in China has become increasingly diversified. Due to the fact that different types of environmental regulation tools have different design principles, their effects on environmental protection governance and corporate business strategies are also different. Therefore, environmental regulation tools have the characteristics of heterogeneity. From the perspective of enterprises, in the face of an increasingly stringent environmental protection system, technological innovation has gradually become an important determinant of balancing environmental regulation compliance costs and business performance, and technological innovation is the core of green innovation (Li et al. 2019) [2]. Due to the continuous changes in the world economic environment and China’s own economic environment, China has adopted different economic policies to promote the healthy development of economy (Yao and Morgan 2008) [3]. Since 2008, governments have begun to adjust their economic policies to alleviate the economic depression caused by the financial crisis. “The Belt and Road”, “Mass entrepreneurship and innovation” and other macroeconomic policies have been introduced by the Chinese government to promote economic recovery and stable development. Enterprises are the main body of innovation and the new force to promote innovation and creation. Improving the technological innovation ability of enterprises is an important guarantee for the sustainable development of enterprises, so the construction of enterprise innovation ability is of great significance (Omri 2020) [4]. The development of high-tech enterprises is more vulnerable to the influence of economic policy, and the change of economic policy is the wind vane of enterprise development strategy. Because of the particularity of the uncertainty of economic policy, it will affect the decision-making behavior of micro enterprises through the uncertain channels of the external environment, and also has a guiding and leading role for the enterprise behavior.

At present, most of the existing studies have analyzed the relationship between environmental regulation and firm innovation from a static perspective, without realizing that the intensity of environmental regulation is not static. Governments change the intensity of environmental regulations according to different economic situations, while firms adjust their coping strategies and their innovation resource allocation in the face of changing environmental regulations. In addition, the different types of environmental regulation tools also determine whether firms adopt short-term production reduction and stress strategies or make long-term innovation investments. Therefore, studying the dynamics of environmental regulation intensity and the impact of environmental regulation heterogeneity on firms’ technological innovation can help us gain a deeper understanding of the mechanism of environmental regulation’s impact on firms’ green innovation. Second, there is much literature on the influence of uncertainty on corporate behavior and the factors affecting corporate technological innovation from all walks of life, but there are few studies on the relationship between economic policy uncertainty, environmental regulation, and corporate technological innovation activities. Compared with the sudden and exogenous nature of other uncertainties, the economic policy uncertainty is more controllable. In this paper, it is significant to study the impact path of economic policy uncertainty in environmental regulation on corporate behavior. The structure of this paper is as follows: the second part introduces the literature review and hypotheses; the third part introduces the data sources and model establishment; the fourth part describes the empirical results and the last part is the conclusion.

The contributions of this paper are as follows: (1) based on the heterogeneity of environmental regulation tools, the research on the incentive effect of external governance mechanisms on corporate innovation. Existing research on environment and innovation mostly focuses on the types of environmental regulations and the impact of environmental regulations on the regional level or industry. In addition, the existing research results are limited to linear results. While considering the differences in the impact of heterogeneous environmental regulation tools, this paper also examines both linear and nonlinear results. Since appropriate environmental regulations can stimulate “innovation compensation” effects, they can not only compensate for firms’ “compliance costs”, but also increase their productivity and competitiveness. However, the greater flexibility of market-based and voluntary environmental regulations can bring about uncertainty. Second, since Porter’s hypothesis originates from developed countries and is not fully applicable to Chinese firms, the linear results will be discussed here along with the nonlinear results; (2) Explore the moderating effects of economic policy uncertainty on different types of environmental regulatory tools on corporate innovation investment. This paper not only studies the independent effect of economic policy fluctuations on innovation output, but also multiplies the independent variables with the moderating variables to determine whether economic policy uncertainty has a moderating effect in the process of environmental regulation and innovation. It is helpful for the government and enterprises to realize greater incentive effect of environmental regulation tools on enterprise innovation in the uncertain environment.

## 2. Literature Review and Research Hypothesis

### 2.1. Literature Review

This paper is related to two branches of literature, firstly a literature review of the Environmental Regulation and Green Innovation and the secondly review of Uncertainty of Economic Policy and Enterprise Innovation Activities.

#### 2.1.1. Environmental Regulation and Green Innovation

Gray (1987) [5], Conrad and Wastl (1995) [6] believe that environmental regulatory policies will increase costs and squeeze innovation resources, thereby hindering enterprise technological innovation. Slater and Ange (2000) [7] consider that when the intensity of environmental regulations is high, the overall R&D level of the enterprise drops, and the benefits of innovation are lower than the costs paid. Many scholars have demonstrated the view that environmental regulation policy restricts enterprise technological innovation from the perspective of empirical test. Nakano (2003) [8] calculated the Malmquist index of the Japanese paper industry and found that environmental regulations did not significantly promote technological innovation. Wagner (2007) [9] and others studied environmental regulation, environmental innovation, and patent application with German manufacturing industry as samples, and found that environmental regulation has a significant negative impact on the number of patent applications, and environmental regulation hindered the green innovation activities of enterprises to a certain extent. Ramanathan (2010) [10] and others used structural equation modeling to analyze the data of the US industrial sector from 2002 to 2006. The study found that in the short term, environmental regulatory policies hindered the technological innovation behavior of enterprises. Kneller and Manderson (2012) [11] found that in the UK manufacturing industry, environmental regulation encourages micro enterprises to carry out environmental innovation, but does not increase the total R & D expenditure. Brunnermeier and Cohen (2003) [12], Hamamoto (2006) [13] believe that appropriate environmental regulations can promote technological progress and diffusion, produce “innovation compensation” effect, and make up for the cost of enterprises complying with environmental regulations. Taking the panel data of industrial enterprises in Taiwan from 1997 to 2003 as samples, it is found that strict environmental regulations can promote the increase of R&D investment of industrial enterprises (Yang et al. 2012) [14]. Greenstone et al. (2012) [15] studied the patent output data of the US manufacturing industry and found that environmental regulations can stimulate technological innovation. Sen (2015) [16] took the automotive industry in a transnational environment as the research object and studied the relationship between environmental regulation and technological innovation, and discovered that environmental regulation can not only improve the level of technological innovation, but also reduce environmental pollution. Yang et al. (2021) [17] found that the strength of environmental regulation was positively related to firms’ green innovation. Moreover, Calel (2011) [18], Bréchet and Meunier (2014) [19] believe that there is a non-linear relationship between environmental regulations and the degree of green technology innovation. Shi et al. (2018) [20] indicated that financial support in government environmental regulations can significantly increase the innovation and scientific research investment of enterprises, and the impact of scientific research investment on enterprise innovation is in an inverted “U” shape. Schmutzler (2001) [21] implies that the mechanism of environmental regulation for innovation compensation is very complicated, and the benefits of innovation may not exceed the cost of complying with environmental regulations. Frondel (2007) [22] represents that the market-incentive environmental regulation policy has no significant impact on the development of pollution end control technology and the innovation of cleaner production technology. Cesaroni (2001) [23] reveals that in a competitive market, the incentive effect of the administrative order-based environmental regulation method on enterprise technological innovation is not as good as the market-incentive environmental regulation method. However, in an imperfectly competitive market, industrial characteristics, economic structure, etc., will affect the effects of different environmental regulatory measures on enterprise technological innovation.

#### 2.1.2. Uncertainty of Economic Policy and Enterprise Innovation Activities

Economic policy uncertainty refers to the inability of economic agents to predict with certainty if, when, and how the government will change current economic policies (Gulen and Ion, 2016) [24]. According to Bloom (2007) [25], the uncertainty of economic policy itself may be an important cause of economic recession. Throughout the existing literature, many scholars agree that the uncertainty of economic policy has a negative impact on the macro-economy. These effects are not only reflected in the rise of economic policy uncertainty, which aggravates the fluctuation of key macroeconomic variables and financial asset variables, but also in the negative impact of economic policy uncertainty on macroeconomic variables such as output and employment, hindering economic recovery (Baker et al. 2016) [26]. These studies suggest that the economic policy uncertainty may inhibit the investment activities of enterprises by changing the cost of business activities, which is related to the characteristics of enterprises and industries such as the proportion of irreversible investment, financial constraints, and the degree of competition. Although many studies at home and abroad focus on the impact of economic policy uncertainty on macroeconomic variables and micro enterprise activities, innovation activities, an important part of economic activities, are ignored by most studies. Bloom (2007) [25] pointed out that although uncertainty will bring temporary negative impact on investment, employment, productivity, and other aspects, due to the difference of adjustment cost characteristics, its impact on R&D may be different from other economic activities. He also indicated that the relationship between uncertainty and R&D activities is a very important topic, which needs more theoretical and empirical research. In addition, Marcus (1981) [27] emphasized that government policies have an important impact on scientific and technological innovation activities. In the face of policy uncertainty, enterprises need to weigh the risks and benefits of innovation activities. This aspect requires more in-depth research. Atanassov et al. (2015) [28] regarded US state elections as an exogenous change of government policy uncertainty. They empirically studied the impact of policy uncertainty on corporate R&D activities and found that rising policy uncertainty led to a rise in corporate R&D levels. At the same time, the positive effect of uncertainty is stronger in competitive election years, politically sensitive industries, enterprises with great difficulty in innovation, enterprises with high growth value and enterprises facing more fierce product market competition. These studies reveal that the impact of policy uncertainty varies with the types of business activities, and its impact on R&D activities is different from that on other types of investment activities. The impact of macroeconomic policy uncertainty on emerging economies is more obvious. As the largest emerging economy, China’s high-tech industrial innovation activities are inevitably affected by the uncertainty of economic policy. In fact, technological innovation activities of enterprises will also be affected by policy uncertainty, and higher economic policy uncertainty has an obvious “incentive effect” on R&D investment of enterprises.

### 2.2. Research Hypothesis

With the continuous development of environmental regulations, environmental regulatory tools are upgraded constantly, environmental regulatory designs are becoming more and more diversified, and the types of regulatory tools continue to grow and develop. Generally speaking, environmental regulations can be divided into three types: command-and-control environmental regulation, market-incentive environmental regulation, and voluntary environmental regulation. Among them, command-and-control environmental regulations use mandatory measures issued by the government to encourage enterprises to fulfill their environmental governance responsibilities. Usually, enterprises have no choice but to abide by rules and regulations passively. In the face of command-and-control environmental regulations, enterprises often take stressful behaviors out of luck, such as temporarily reducing production and other measures to reduce corporate emissions and ensure temporary environmental compliance. In addition, the environmental regulation system in China is under the joint leadership of the government at the same level and higher level organizations, so the enterprises will be affected by the improper performance view and excessive intervention of local governments, which leads to the low status of environmental regulation departments, lack of independence, and the effective implementation of environmental regulation policies (Tang et al. 2010) [29].

Based on this, this paper argues that the command-and-control environmental regulation does not significantly promote the technological innovation activities of enterprises, and even has a “crowding out effect” on innovation investment. The main reasons are as follows: first, the command-and-control environmental regulation often has the characteristics of high cost, and the regulated enterprises often need to meet the pollution control standards through high-cost pollution control means. When the enterprises are short of funds, they may use the funds originally used for innovation to pay the pollution fee, or even spend more resources to deal with the environmental regulation policies for the temporary treatment of pollutants, so there may be a crowding out effect on the technological innovation investment of enterprises. Second, the high cost of command-and-control environmental regulation increases the production cost of enterprises, which may have an adverse impact on the profits of enterprises, further reduces the limited resources of enterprises, and then reduces the willingness of enterprises to carry out technological innovation activities.

**Hypothesis** **1a** **(H1a).** *Compared with command-and-control environmental regulatory tools, market-incentive environmental regulatory tools and voluntary environmental regulatory tools have a more significant positive impact on the enterprise’s green innovation output.*

Second, since the Porter hypothesis suggests that appropriate environmental regulations can stimulate “innovation compensation” effects, which not only compensate for the “compliance costs” of firms, but also increase their productivity and competitiveness. However, when the Porter hypothesis was developed in developed countries such as the United States and tested directly in developing countries such as China, the premise of the theory underwent a fundamental structural change and, therefore, may not fully reflect the laws of developing countries such as China. Moreover, there is uncertainty about the effect of environmental regulation on technological innovation, so a U-shaped relationship between market-incentive environmental regulation and technological innovation is considered. Therefore, the hypothesis is put forward:

**Hypothesis** **1b** **(H1b).** *Market-incentive environmental regulation tools have an inverted U relationship with green innovation output.*

In recent years, more and more scholars have proposed that economic policy uncertainty will promote enterprise innovation output. When faced with the increase of economic uncertainty, enterprises tend to choose the growth option of innovation investment. Because the higher the uncertainty of economic policy, the greater the possibility of disruptive changes in the market, the greater the opportunity for enterprises to obtain future competitive advantage, and the greater the possibility for enterprises to obtain future growth opportunities through early innovation investment. Vo and Le (2017) found that due to the significant positive correlation between R&D investment and the improvement of corporate competitiveness, in order to maintain sustained competitiveness, enterprises will increase R&D investment to cope with the negative impact of increased economic policy uncertainty on enterprises. The research of Ross et al. (2018) [30] also shows that the increase in economic policy uncertainty stimulates R&D investment. Therefore, this article proposes:

**Hypothesis** **2** **(H2).** *Economic policy uncertainty has a positive impact on green innovation output.*

The uncertainty of macroeconomic policy brings more uncertainty to the development of enterprises, and also affects the implementation of micro environmental regulation policy. Innovation is an important driving force of economic growth. Enterprises with strong innovation ability can obtain strong market power and higher excess profits. When faced with market competition and risk, enterprises tend to accelerate innovation to increase market power to a certain extent (Aghion et al. 2015) [31]. Meanwhile, the increase of economic policy uncertainty may aggravate the market risk, which will make enterprises further increase innovation investment to maintain or regain market power. Therefore, under different environmental regulation policies, the regulation effect of economic policy uncertainty is also different. First of all, market-incentive environmental regulation is a more flexible policy, and is more affected by market factors. When enterprises face external economic policy uncertainty, it will make the U-shaped relationship between market-oriented environmental regulation and innovation output more obvious. In other words, when enterprises are faced with voluntary environmental regulation, the increase of economic policy uncertainty may aggravate the market risk, which will make enterprises further reduce innovation investment to keep the enterprise itself. Therefore, this paper puts forward the following suggestions

**Hypothesis** **3a** **(H3a).** *When enterprises are subject to Market Incentive Environmental Regulation, economic policy uncertainty positively moderates the inverted U-shaped relationship between environmental regulation and enterprise innovation output.*

**Hypothesis** **3b** **(H3b).** *When enterprises are subject to Voluntary Environmental Regulation, economic policy uncertainty negatively regulates the relationship between environmental regulation and enterprise innovation output.*

**Figure 1:**
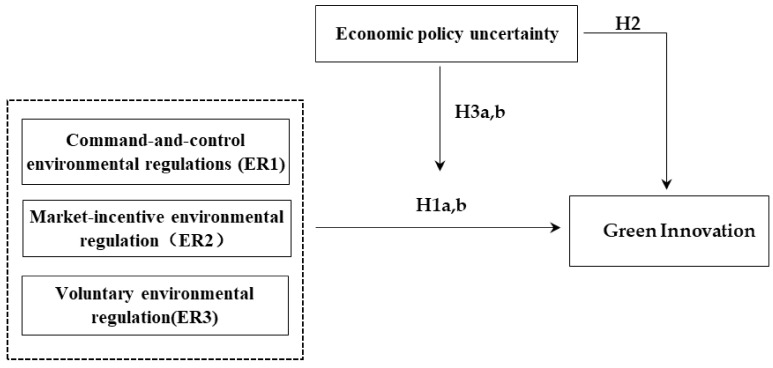
Research design.

The technical roadmap of this paper is as follows (Figure 1):

## 3. Research Design

### 3.1. Sample Selection and Data Sources

Regarding the sample selection of high-tech enterprises, the paper considers that listed enterprises only began to regulate the disclosure of R&D investment in 2008, and the certification standards for high-tech enterprises were officially implemented in 2008. Taking into account the immaturity and irregularity of the certification measures and R&D intensity from 2008 to 2011, and the lack of data is more serious. In this section, we use panel data from 2012 to 2017 for empirical research. Through the analysis of the advantages and disadvantages of previous scholars’ sample selection methods, based on the considerations of accuracy and cost, we propose a more reasonable sample selection method for high-tech enterprises. First, the 2008–2017 stock code data of all Chinese A-share listed enterprises are derived from the CSMAR database and matched with the CSMAR qualification database to determine the parent enterprise and subsidiary as the sample data of high-tech enterprises. Second, according to the “Administrative measures for the determination of high and new technology enterprises”, if it is recognized as a high-tech enterprise, the income tax discount it can enjoy is 15%, and the validity period is 3 years. Then, we used CSMAR’s corporate tax rate data to multiple cross-check the sample data during the three years after the high-level enterprise identification to determine whether the enterprise still enjoys the 15% tax preference, so as to further determine the completeness and accuracy of the sample selection. If there is a mismatch, manually collect the annual report and official website data for comparison, and finally determine the appropriate high-tech enterprise sample. Because some listed enterprises did not disclose the certification of high-tech enterprises in the annual reports, or disclosed that they were recognized as high-tech enterprises but the subsequent annual reports did not disclose whether they passed the review, or did not disclose that they did not apply for review, failed the review or were revoked as high-tech enterprises matter, which will affect whether a listed enterprise has the qualifications of a high-tech enterprise, which is very important for the research samples and research conclusions in the paper. Therefore, in addition to sorting out the samples listed in the qualification accreditation database, the paper also further determines the high-tech enterprise qualifications of the samples in combination with the local publicity documents on the “High-tech Enterprise Accreditation Management Network” to ensure that the data are true and reliable.

### 3.2. Data Source

The number of environmental protection laws and regulations in force from all regions of the year, the amount of sewage charges remitted and put into storage in different regions, the number of households paid and put into storage in each region, the number of environmental letters and visits in each region are from “China Environment Yearbook”. However, the environmental petition data of each region is only disclosed to 2015. In order to ensure the comparability of the sample interval, this paper makes up for the missing data of environmental petition in 2016 and 2017 through the data of environmental petition in 2015 and the rising rate of environmental letters and visits disclosed in “China Environmental Yearbook”. The main financial data comes from the CSMAR database and the CCER database. Moreover, the sample of high-tech enterprises comes from the Cathay Certified Qualification Database and Tax Rate Database. This paper mainly uses EXCEL2019 and Stata15.0 software for data processing and statistical analysis. After screening, this paper obtained 3438 panel observations from 573 sample enterprises.

### 3.3. Variable Definition

This section contains the definition of variables, mainly the choice of environmental regulation variables and the choice of green innovation variables for firms, as well as the choice of moderating and control variables.

#### 3.3.1. Selection of Environmental Regulation Tool Variables

Based on the research of Shen et al. (2019) [32], this paper measures the three kinds of environmental regulation, divides the environmental regulation into command control type, market incentive type and voluntary type. On this basis, it constructs an index system to evaluate the regional industrial competitiveness, empirically analyzes the impact of the heterogeneity of environmental regulation tools on the regional industrial competitiveness, and tests the spatial effect of different types of environmental regulation.

First, command-and-control environmental regulation tool refers to the administrative department’s direct management and mandatory supervision of environmental related production activities according to relevant laws, regulations, rules, and standards. Governments, industry organizations, and environmental protection departments have formulated a variety of environmental protection systems and standards to control environmental pollution sources by setting the lower limit of environmental protection and putting environmental protection matters in front. Since the stringency of the regulatory system can vary at different levels, the use of quantitative measures also takes into account the stringency of the policy. Therefore, this paper uses the current effective environmental regulations and rules to measure the command-and-control environmental regulation tool (ER-1).

Second, the target of market incentive environmental regulation mainly exists in the form of tax preference, while the market incentive environmental regulation system in China is not perfect. Comparatively speaking, the pollution discharge fee system was implemented earlier and the tool implementation was relatively stable, which can effectively measure the cost of corporate pollution control. Therefore, this paper selects the ratio of the amount of sewage charges collected by each province, autonomous region, and municipality to the industrial added value (unit: 10,000 yuan/household) as an indicator for evaluating market-incentive environmental regulations (ER-2).

Lastly, this paper selects the data of voluntary supervision at the regional level as an alternative indicator to measure the intensity of voluntary environmental regulation (ER-3). Specifically, the logarithm of the total letters received in each region is used as an alternative indicator of voluntary environmental regulation tools.

#### 3.3.2. Variable Selection of Enterprise Green Innovation

In order to screen green patents of listed enterprises, this paper is based on the green patent data, which is compared with the international patent classification green list launched by WIPO in the State Intellectual Property Office. This list is generated according to the classification standard of green patents in the United Nations Framework Convention on Climate Change, including seven categories: Transportation, Waste Management, Energy Conservation, Alternative Energy Production, Administrative Regulatory or Design Aspects, Agriculture or Forestry, and Nuclear Power Generation. At the same time, in order to further reflect the innovation and value of green patents, this paper presents the green invention patent (Gpatent) to represent the green innovation output.

#### 3.3.3. Moderator Variable

In order to measure the economic policy uncertainty, Baker et al. (2016) [26] constructed the measurement index of economic policy uncertainty. This indicator was developed in 2016 based on keyword searches in the English-language articles of the South China Morning Post in Hong Kong, China, and the specific development process was as follows: first, the articles were extracted from the monthly articles that contained both “China”, “economic” and “uncertain” keywords; second, the above extracted articles were deeply screened, and the screened articles included at least one of the following keywords such as “Spending”, “Policies”, “Tax”, “Central bank”, “Budget”, “Deficit”, etc. “Finally, the ratio between the number of articles extracted after two screening and the total number of articles in the South China Morning Post for that month was calculated to obtain monthly data measuring the degree of uncertainty in China’s economic policies. In this paper, the ln(epu) of monthly average is taken as the alternative variable of economic policy uncertainty.

#### 3.3.4. Control Variable

The paper first controls the degree of marketization. In addition, enterprise innovation investment is affected by enterprise profitability, risk-taking, and other factors. Therefore, this paper controls the micro enterprise level variables such as return on assets, solvency, profitability, management incentive, tax rate, and so on. See Table 1 for the definition of specific variables.

### 3.4. Model Construction

In order to test the impact of different environmental regulation tools on green innovation output, the paper constructs model 1. Model 1 verifies the impact of different environmental regulatory tools on the green innovation output of enterprises. Meanwhile, considering the time lag of innovation, the explained variable in model 1 include the current period and the lag period.

(1) $$GPatent_{i,t}/GPatent_{i,t+1}=\beta_{0}+\beta_{1}ER_{i,t}+\beta_{2}M-Index_{i,t}\;+\beta_{3}LEV_{i,t}+\beta_{4}NPM_{i,t}+\beta_{5}TAX_{i,t}+\beta_{6}Dual_{i,t}+\beta_{7}Indir_{i,t}+\beta_{8}S_{i,t}+\beta_{9}LnMS_{i,t}+\beta_{10}ROA_{i,t}+\varepsilon$$

- Model 1

In order to further test whether there is an inverted U-shaped relationship between market-incentive environmental regulation and green innovation output, this paper adds the square term of ER-2 to characterize the inverted U-shaped effect.

(2) $$GPatent_{i,t}=\beta_{0}+\beta_{1}ER_{i,t}+\beta_{2}ER_{i,t}*ER_{i,t}+\beta_{3}M-Index_{i,t}+\beta_{4}LEV_{i,t}+\beta_{5}NPM_{i,t}+\beta_{6}TAX_{i,t}\;+\beta_{7}Dual_{i,t}+\beta_{8}Indir_{i,t}+\beta_{9}S_{i,t}+\beta_{10}LnMS_{i,t}+\beta_{11}ROA_{i,t}+\varepsilon$$

- Model 2

In order to test the impact of the fluctuation of economic policy uncertainty on enterprise innovation output, this paper constructs model 3.

(3) $$GPatent_{i,t}=\beta_{0}+\beta_{1}lnepu_{i,t}++\beta_{2}M-Index_{i,t}+\beta_{3}LEV_{i,t}+\beta_{4}NPM_{i,t}+\beta_{5}TAX_{i,t}+\beta_{6}Dual_{i,t}\;+\beta_{7}Indir_{i,t}+\beta_{8}S_{i,t}+\beta_{9}LnMS_{i,t}+\beta_{10}ROA_{i,t}+\varepsilon$$

- Model 3

In order to test the moderating role of economic policy uncertainty in environmental regulation and innovation output, this paper constructs models 4 and 5.

Model 4 examines the moderating effect of economic policy uncertainty on the U-shaped relationship between market-incentive environmental regulations and innovation output.

(4) $$GPatent_{i,t}=\beta_{0}+\beta_{1}ER_{i,t}+\beta_{2}ER_{i,t}*ER_{i,t}+\beta_{3}ER_{i,t}*ER_{i,t}*Lnepu_{i,t}+\beta_{4}M-Index_{i,t}\;+\beta_{5}LEV_{i,t}+\beta_{6}NPM_{i,t}+\beta_{7}TAX_{i,t}+\beta_{8}Dual_{i,t}+\beta_{9}Indir_{i,t}+\beta_{10}S_{i,t}+\beta_{11}LnMS_{i,t}+\beta_{12}ROA_{i,t}+\varepsilon$$

- Model 4

Model 5 examines the moderating effect of economic policy uncertainty on the relationship between command-and-control, voluntary environmental regulations, and innovation output.

(5) $$\;\;\;\;\;\;\;GPatent_{i,t}=\beta_{0}+\beta_{1}ER_{i,t}+\beta_{2}Lnepu_{i,t}+\beta_{3}ER_{i,t}*Lnepu_{i,t}+\beta_{4}M-Index_{i,t}+\beta_{5}LEV_{i,t}+\beta_{6}NPM_{i,t}\;+\beta_{7}TAX_{i,t}+\beta_{8}Dual_{i,t}+\beta_{9}Indir_{i,t}+\beta_{10}S_{i,t}+\beta_{11}LnMS_{i,t}+\beta_{12}ROA_{i,t}+\varepsilon$$

- Model 5

In models 1 to 5, *β*_0_ is the intercept, *β*_1_~*β_n_* is the coefficient (*n* = 1, 2, …), *ε* the residual.

## 4. Empirical Results

### 4.1. Descriptive Statistics

Table 2 shows the descriptive statistical results of enterprise green innovation output, heterogeneity of regional environmental regulation tools moderator variable, and control variables.

It can be seen from Table 2 that the average value of the full sample of green technology innovation output is 0.579, and the maximum and minimum values are 65 and 0, respectively. From this point of view, it can be seen that there is a large gap in the innovation input and output of different enterprises. Moreover, most enterprises are in a state of low input and low output. Furthermore, the mean value of ER-1 is 33.778, which is less than the median of 35. The maximum value is 105 and the minimum value is 3. The average ER-2 is 6.272, and the standard deviation of the whole sample is 0.045. The average of ER-3 is 8.588, the minimum is 4.7, and the maximum is 10.077. Therefore, different types of environmental regulations have great regional differences. Furthermore, the maximum value of economic policy uncertainty is 5.902, and the minimum value is 4.744, which indicates that the fluctuation of economic policy uncertainty is small during 2012–2017.

### 4.2. Empirical Results

This paper selects balanced panel data for analysis and uses the Hausman test to select the model. The *p* value of Hausman test rejects the null hypothesis at the 1% significance level, that is, the fixed-effects model is the most efficient. Therefore, the empirical model of this paper uses the fixed-effects model for regression as well. This article uses stata15.0 to perform firm-level clustering standard error fixed-effects model regression on sample data.

### 4.3. Analysis on the Impact of the Heterogeneity of Environmental Regulation Tools on Enterprise Green Innovation

When studying the influence system of different environmental regulation tools on the green innovation output of enterprises, this paper first analyzes the influence of different types of environmental regulation tools on the enterprise innovation output (GPatent-Model 1). Table 3 lists (1)–(3) the regression results of the model panel.

Through the regression results of model 1 in Table 3, it can be seen that market-incentive environmental regulation (ER-2) and voluntary environmental regulation (ER-3) have a significant positive impact on enterprise innovation output. Moreover, Jiang et al. (2020) [33] drew the same conclusion when studying voluntary environmental regulation. At the same time, the incentive effect of voluntary environmental regulation tools on enterprise innovation output is more significant. However, Er-1 does not significantly increase innovation output. This may be because when facing the command-and-control type of environmental regulation, enterprises may spend more money on pollution control, which has reached the government control standard, and they have not fundamentally solved the pollution problem based on the consideration of technological innovation.

From the regression results of model 2, it can be seen that the coefficients of the first and second power terms are significantly positive and negative at the level of 1%, respectively. This indicates that there is a significant inverted U-shaped curve relationship between market-incentive environmental regulation and innovation output, which means that there is an inflection point between market-incentive environmental regulation and innovation output, and the inflection point is 15.67. The result is consistent with the results of Pan et al. (2021) [34] and Song et al. (2020) [35]. Specifically, when the intensity of environmental regulation in an area is less than the threshold, the enhancement of the intensity of environmental regulation promotes the increase of enterprise innovation output. At this time, the effect of “innovation compensation” is greater than the effect of “following cost”, which reflects the Porter hypothesis effect. Moreover, when the intensity of regulation is greater than the threshold, the inhibitory effect of environmental regulation on the innovation output of the enterprise takes the upper part, and the “innovation compensation” effect cannot effectively compensate for the “compliance cost” effect, which reflect the neo-classical environmental regulation “restraint theory”.

### 4.4. Analysis of the Impact of Economic Policy Uncertainty on Enterprises’ Innovation Output

When studying the impact of economic policy uncertainty on green innovation output, this chapter first studies and analyzes the impact of economic policy uncertainty (lnepu) on green innovation output (Gpatent), and then studies the regulatory role of economic policy uncertainty. Table 4 shows the panel regression results of model 4 and 5.

#### 4.4.1. Analysis of the Transmission Mechanism of Economic Policy Uncertainty to Green Innovation

From the panel data regression results of model 3 in Table 4, it can be concluded that economic policy uncertainty has a significant impact on enterprise innovation output, which is the same as that of Shen et al. (2020) [32]. To be specific, the result shows that when the economic policy uncertainty increases, high-tech enterprises will increase investment in innovation to save enterprise opportunities and improve enterprise competitiveness. It also shows that Chinese high-tech enterprises are actively coping with the fluctuations of economic policies. Moreover, in the case of strong economic policy uncertainty, enterprises tend to use innovation to resolve market risks and seize market advantages, and innovation has produced better economic and environmental benefits. Thus, the adjustment of economic policy can promote enterprise innovation output.

#### 4.4.2. The Adjustment Effect of Economic Policy Uncertainty on the Main Effect

The moderating effect of economic policy uncertainty on the relationship between environmental regulation and innovation output is shown in Table 4 list (6), (7). Because command-and-control environmental regulations have no significant impact on the innovation output of enterprises, we will not test the moderating effect below.

From Table 4 list (6), we can find that economic policy uncertainty positively regulates the relationship between market-incentive environmental regulations (ER-2) and innovation output, which shows that when economic policy uncertainty is higher, the U-shaped relationship between the two is more obvious. Furthermore, it shows that market-incentive environmental regulations are affected by external factors.

In list (7), we find that economic policy uncertainty negatively regulates the relationship between voluntary environmental regulation and innovation output. This shows that when enterprises are subject to voluntary environmental regulation (ER-3), economic policy uncertainty negatively regulates the path of innovation output. This might be voluntary environmental regulation and the behavior of the enterprise’s own behavior. When facing the external environment with higher risk, the enterprise will reduce its own risk to ensure its long-term development. Therefore, enterprises may develop more businesses that require less risk, instead of investing a lot of money to engage in technological innovation, which further shows that flexible environmental regulation tools are more conducive to the realization of incentives for corporate innovation. Overall, within the limits of environmental pollution discharge, flexible environmental policies encourage enterprises to adopt environmentally friendly technologies, coordinate their green production behaviors through reward and punishment mechanism, promote enterprises’ green management, help enterprises establish a good corporate image, and encourage enterprises to carry out technological innovation.

#### 4.4.3. The Lag Effect of Environmental Regulation on Innovation Effect

Due to the time lag of innovation, we consider the lagged effect of environmental regulation on innovation output. Since some of the data are only available from 2012 to 2017, the independent variables are selected from 2012 to 2016 to explore the lagged effects. The main data results are presented in Table 5. The result of the lag effect is basically consistent with the result of the non-lag data.

## 5. Conclusions

The paper uses high-tech enterprises from 2012–2017 as a research sample to empirically test the impact of different environmental regulatory tools on corporate green innovation. At the same time, it studies the impact of economic policy uncertainty on corporate green innovation, and further explored the moderating effect of economic policy uncertainty on the relationship between environmental regulation and enterprise innovation activities. First of all, in the research of the paper, command-and-control environmental regulation tools does not have a significant impact on green innovation because they only set a lower limit for environmental protection, which does not provide enough incentive for green innovation and may even have the effect of “driving out good money from bad money” on the level of environmental protection of the whole society. Moreover, the sudden increase in environmental technology standards may force companies to stop their existing investment projects and have a crowding-out effect on innovation resources. Second, both market-incentive environmental regulations and voluntary environmental regulations have a significant positive impact on the green innovation output of enterprises. Furthermore, there is a significant inverted U-curve relationship between market-incentive environmental regulations and green innovation output. When market-based environmental regulations are small, the “compliance cost effect” is stronger, and environmental regulations are not conducive to green innovation; when market-based regulations are large, the “innovation compensation effect” is stronger, and environmental regulations are conducive to green innovation. This also shows that the market-incentive environmental regulation is more flexible than the voluntary environmental regulation, giving enterprises more free choice, and promoting the green innovation output of enterprises. Additionally, economic policy uncertainty is positively promoting the enterprises green innovation output. Second, when economic policy uncertainty adjustment is used as a moderating variable, it positively regulates the u-shaped relationship between market-incentive environmental regulations and corporate green innovation. However, the economic policy uncertainty has weakened the relationship between voluntary environmental regulations and enterprises green innovation. Therefore, the interactive effect of economic policy uncertainty and different types of environmental regulatory tools shows that when enterprises face more flexible regulatory tools, the impact of economic policy uncertainty on enterprises will be more sensitive, which also shows that it is more conducive to the development of enterprise innovation activities, and adjust enterprise innovation strategies according to market volatility.

### 5.1. Suggestion

First, we should continue to attach importance to the importance of environmental protection and adhere to the sustainability of environmental protection. In the “high-quality” development stage of China’s economy, environmental protection is still an important task in the government work. According to research results, environmental regulations can stimulate enterprises’ innovative activities or behaviors. In the face of the government’s mandatory regulation policies, high-tech enterprises tend to respond to the government’s environmental regulations by making use of their independent research. On the other hand, the technological innovation caused by environmental regulations can significantly improve the business performance of enterprises in the short term. In the long run, technological innovation is the decisive factor for the improvement of competitiveness enterprises in the future. Moreover, environmental problems are rooted in social progress, and there is always a dilemma between environmental protection and development. Furthermore, short-term environmental protection effects achieved through strict regulations are not desirable. Accordingly, our government should continue to attach importance to environmental protection, and fully mobilize the enthusiasm of enterprise innovation through effective regulation policies to realize the sustainability of environmental protection and development.

Second, improve the performance assessment system for local governments and implement a consistent, stable and transparent environmental regulation policy. At present, the central government is strengthening environmental regulations, but local governments are constantly adjusting the intensity of environmental regulations due to the interests of regional economic growth, so there are large fluctuations in the intensity of environmental regulations in each region, and the uncertainty of environmental regulations is high. To avoid the impact of uncertainty on enterprises, the government should establish a more scientific and reasonable performance appraisal system, incorporate environmental protection and governance indicators into local government performance appraisals, and establish methods for investigating major environmental accidents. This will prevent local governments from constantly adjusting the intensity of environmental regulations in the game of interests with the central government, which will undermine the incentive of enterprises to innovate in technology. At the same time, the government should advocate the implementation of consistent, stable, and transparent environmental regulation policies, improve the transparency of environmental regulation policies, collect opinions from society before introducing or changing policies, allow sufficient time for enterprises to receive information, and pay attention to the impact of the external environment of economic policy uncertainty on enterprises to avoid the risks of enterprise innovation brought about by environmental regulation uncertainty.

### 5.2. Limitation

This paper investigates the impact of environmental regulation tools on firms’ green innovation activities and reveals the realization path of environmental regulation to enhance innovation capability in a cross-level context. However, the research in this paper still suffers from the following shortcomings. Based on the availability of data, this paper only analyzes explicit environmental regulations, but not the impact of implicit environmental regulations on firms’ technological innovation activities. At the same time, this paper selects specific environmental regulation policy implementation statistics to measure different types of environmental regulation tools, however, the effectiveness of environmental regulation tools is difficult to be reflected by a single policy implementation effect indicator, so how to better study the impact of different types of environmental regulation tools on firms’ technological innovation activities remains to be further discussed.

## Figures and Tables

**Table 1:** The definition of variables.

Category	Variable Name	Variable Symbol	Index Calculation
Explained variable	Technological innovation output	GPatent	Granted amount of green invention patents by enterprises
Explanatory variables	Command-and-control environmental regulations	ER-1	Number of current effective environmental protection laws and regulations in each region
	Market-incentive environmental regulation	ER-2	The amount of sewage charges paid into the warehouse/the number of households that have been paid into the warehouse
	Voluntary environmental regulation	ER-3	The logarithm of the total environmental letters received and visits in each region
Moderator	Economic policy uncertainty	Lnepu	The arithmetic average method is transformed into logarithm of annual data
Control variable	Marketization index	M-Index	Fan Gang et al. (2016) in the market index system “Overall Score of Marketization Process”
	Return on Assets	ROA	Ratio of total liabilities to total assets
	Assets and liabilities	LEV	Net profit/total assets
	Comprehensive tax rate	TAX	(Business taxes and surcharges + income tax expenses)/Total operating income
	Two jobs in one	Dual	Whether the chairman and general manager are the same person, is it 1, if it is 0
	Operating net profit margin	NPM	Net profit/operating income
	Proportion of independent directors	Indir	Number of independent directors/number of directors
	Equity checks and balances	S	The sum of the equity ratio of the second largest shareholder to the tenth largest shareholder /the shareholding ratio of the first largest shareholder
	Management incentives	lnMS	Take the logarithm of the total annual salary of directors, supervisors and senior management

**Table 2:** Descriptive statistical results.

Variable	Obs	Mean	Std. Dev.	Min	Max	Median
GPatent	3432	0.579	3.166	0	65	0
ER-1	3438	33.778	19.011	3	105	35
ER-2	3438	6.272	4.05	1.515	33.994	5.471
ER-3	3438	8.588	0.843	4.7	10.077	8.701
Lnepu	3438	5.347	0.464	4.744	5.902	5.354
M-Index	3438	8.313	1.578	2.87	10.29	8.89
LEV	3438	0.427	0.185	0.008	0.979	0.418
NPM	3438	0.063	0.236	−8.911	2.024	0.058
TAX	3438	0.024	0.03	−0.316	0.774	0.019
Dual	3438	0.273	0.446	0	1	0
Indir	3438	0.369	0.053	0.25	0.714	0.333
S	3438	0.836	0.741	0.015	8.173	0.675
LnMS	3438	15.304	0.685	13.045	18.772	15.258
ROA	3438	0.042	0.055	−0.448	0.361	0.036

**Table 3:** Empirical results.

Variables	(1)	(2)	(3)	(4)
	Model 1			Model 2
	Gpatent			Gpatent
ER-1	0.005			
	(1.51)			
ER-2		0.054 **		0.226 **
		(2.47)		(2.34)
ER-2^2^				−0.007 **
				(−2.21)
ER-3			0.299 *	
			(1.70)	
M-Index	0.103	0.036	0.036	0.011
	(0.58)	(0.20)	(0.19)	(0.06)
LEV	0.135	0.140	0.125	0.202
	(0.23)	(0.23)	(0.21)	(0.33)
TAX	1.284	1.294	1.408	1.191
	(0.78)	(0.81)	(0.88)	(0.77)
ROA	−2.119 *	−1.896 *	−2.133 *	−1.836 *
	(−1.93)	(−1.73)	(−1.94)	(−1.68)
Dual	−0.128	−0.150	−0.130	−0.145
	(−1.42)	(−1.62)	(−1.43)	(−1.57)
NPM	0.114	0.091	0.114	0.108
	(1.62)	(1.27)	(1.54)	(1.60)
Indir	−0.217	−0.346	−0.124	−0.416
	(−0.15)	(−0.25)	(−0.09)	(−0.30)
S	−0.178	−0.182	−0.189	−0.186
	(−1.02)	(−1.03)	(−1.07)	(−1.05)
LnMS	0.338 *	0.284	0.314 *	0.215
	(1.69)	(1.62)	(1.75)	(1.35)
Constant	−5.348 **	−4.098 **	−6.855 **	−3.515 *
	(−2.14)	(−1.98)	(−2.30)	(−1.87)
Observations	3432	3432	3432	3432
Number of std	572	572	572	572
R-squared	0.006	0.008	0.007	0.012

Robust t-statistics in parentheses ** *p* < 0.05, * *p* < 0.1.

**Table 4:** Empirical results.

Variables	(5)	(6)	(7)
	Model 3	Model 4	Model 5
	Gpatent	Gpatent	Gpatent
ER-2		0.217 **	
		(2.18)	
ER-2^2^		−0.020 ***	
		(−2.85)	
ER-3			1.393 *
			(1.78)
lnc	0.290 ***	0.056	2.100 *
	(3.08)	(0.49)	(1.88)
ER-2^2^ × lnc		0.002 **	
		(2.32)	
ER-3 × lnc			−0.217 *
			(−1.69)
M-Index	−0.010	−0.017	−0.016
	(−0.06)	(−0.10)	(−0.10)
LEV	0.227	0.261	0.236
	(0.38)	(0.43)	(0.40)
TAX	0.657	0.538	0.872
	(0.45)	(0.36)	(0.58)
ROA	−1.959 *	−1.696	−1.843 *
	(−1.87)	(−1.58)	(−1.76)
Dual	−0.123	−0.142	−0.129
	(−1.37)	(−1.55)	(−1.43)
NPM	0.110	0.094	0.083
	(1.52)	(1.33)	(1.11)
Indir	−0.212	−0.440	−0.198
	(−0.15)	(−0.32)	(−0.14)
S	−0.190	−0.187	−0.183
	(−1.06)	(−1.03)	(−1.03)
LnMS	0.268	0.184	0.248
	(1.43)	(1.13)	(1.38)
Constant	−4.766 **	−3.011	−16.086 *
	(−2.04)	(−1.63)	(−1.96)
Observations	3,432	3,432	3,432
Number of std	572	572	572
R-squared	0.009	0.012	0.009

Robust t-statistics in parentheses; *** *p* < 0.01, ** *p* < 0.05, * *p* < 0.1.

**Table 5:** Empirical results.

Variables	(1)	(2)	(3)
		Model 1	
	GPatent_lag	GPatent_lag	GPatent_lag
ER1	0.007		
	(1.21)		
ER2		0.108 ***	
		(3.55)	
ER3			0.390 ***
			(2.59)
M-Index	0.154	0.095	0.125
	(1.46)	(0.89)	(1.18)
LEV	−0.030	0.027	−0.045
	(−0.05)	(0.04)	(−0.07)
TAX	−0.643	−1.174	−0.551
	(−0.20)	(−0.36)	(−0.17)
ROA	0.354	0.916	0.479
	(0.22)	(0.57)	(0.30)
Dual	−0.144	−0.166	−0.154
	(−0.78)	(−0.90)	(−0.83)
NPM	−0.035	−0.091	−0.048
	(−0.14)	(−0.35)	(−0.18)
Indir	−0.306	−0.518	−0.210
	(−0.20)	(−0.34)	(−0.14)
S	−0.342 **	−0.343 **	−0.354 **
	(−2.35)	(−2.36)	(−2.43)
LnMS	0.399 **	0.283	0.379 **
	(2.28)	(1.59)	(2.16)
Constant	−6.506 **	−4.598 *	−9.071 ***
	(−2.50)	(−1.74)	(−3.26)
Observations	2860	2860	2860
Number of std	572	572	572
R-squared	0.008	0.013	0.010

Robust t-statistics in parentheses; *** *p* < 0.01, ** *p* < 0.05, * *p* < 0.1.

## Data Availability

The data presented in this study are available on request from the corresponding author.
